# Predictors of Poor Outcomes for COVID-19-Associated Pneumonia in a Minority Population

**DOI:** 10.7759/cureus.12431

**Published:** 2021-01-02

**Authors:** Ibrahim Omore, Idayat Brimah, Sulaiman Tijani, Abimbola Fadairo-Azinge, Melissa Gazi, Ismail O Malik, Padmaja Sajja, Abdulla M. Ali, Raji Ayinla, Hussein Assallum

**Affiliations:** 1 Internal Medicine, Harlem Hospital Center, New York, USA; 2 Public Health, University of Alabama at Birmingham, Birmingham, USA; 3 Pulmonary and Critical Care Medicine, Harlem Hospital Center, New York, USA

**Keywords:** covid-19, predictors, minority population

## Abstract

Background

In December 2019, an unprecedented outbreak of pneumonia of unknown etiology emerged in Wuhan City, Hubei province in China. A novel coronavirus was identified as the causative agent and was subsequently termed COVID-19 by the World Health Organization (WHO). It rapidly became a pandemic, and it has been a significant challenge to healthcare providers to predict outcomes of the infected patients.

Objective

The aim of this study was to investigate the clinical characteristics of patients admitted for COVID-19 infection in an Inner-City Hospital in New York City, to assess the correlation between inflammatory markers and outcomes prediction in a high-risk population.

Methods

We identified 235 patients who were admitted to our Hospital in NYC between March 19th and April 25th, 2020 with laboratory confirmed COVID-19 diagnosis with associated pneumonia and who also had documented inflammatory markers (D-dimer, C-reactive protein, lactate dehydrogenase, ferritin, procalcitonin) during their hospital stay.

Results

The study population was predominantly non-Hispanic black. There was no statistically significant difference between survivors and non-survivors by race and/or ethnicity (P = 0.69). Thirty-five percent of the patient population had died by the end of this study and those that died had a higher mean age compared to survivors (69.5 ± 13.6 vs 63.8 ± 15.2, P = 0.004). There is a significant difference in the D-dimer levels in patients who survivedwhen compared to those who died (P = 0.002). A higher proportion of patients that died were admitted to the ICU, (23.7% vs 55.4%, P < 0.0001) and/or intubated (18.4% vs 51.8%, P < 0.0001).

Conclusion

Our study demonstrated that patients who died had a significantly higher D-dimer (>3,000) when compared with survivors. Higher mean age was associated with increased mortality and admission to ICU and/or intubation.

## Introduction

Since the occurrence of Severe Acute Respiratory Syndrome Coronavirus 2 (SARS-CoV-2) in late 2019, many theories have been advanced about how severe Coronavirus Disease - 2019 (COVID-19) induces Acute Respiratory Distress Syndrome (ARDS) with subsequent high morbidity and mortality. It is believed that COVID-19 may predispose patients to thrombotic disease both in the venous and arterial circulations due to excessive inflammation, platelet activation, endothelial dysfunction, and stasis [1]. Although most symptomatic patients with COVID-19 predominantly have a respiratory tract infection, a proportion of patients progress to a more severe and systemic disease, characterized by treatment-resistant pyrexia, acute lung injury with acute respiratory distress syndrome, shock, and multiple organ dysfunction, which is associated with substantial mortality [2]. Many patients with severe COVID-19 pneumonia present with coagulation abnormalities that mimic other systemic coagulopathies associated with severe infections, such as disseminated intravascular coagulation (DIC) or thrombotic microangiopathy (TMA); however, COVID-19 has distinct features [3]. Coagulopathy in patients with COVID-19 pneumonia is associated with an increased risk of death [4]. Most studies involving inflammatory markers and outcomes in patients with COVID-19 have looked at only patients with severe disease. While there are no standardized criteria for defining severity of disease, some scores like Brescia-COVID Severity scale have been designed to help clinicians in recognizing severe cases, none of these scoring systems have been validated [5]. This study looked at the clinical characteristics of all admitted patients, who were COVID-19 polymerase chain reaction (PCR) positive in an Inner-City Community Hospital in NYC between March 19th and April 25th, 2020 irrespective of the severity of the disease. The aim of this study is to identify contributing factors to higher mortality of COVID-19 associated pneumonia among minority populations.

In this study, we defined severe disease as patients with COVID-19 who require intubation or ICU care during their hospitalization.

## Materials and methods

This is a retrospective observational single-center study of patients admitted with COVID-19 infection between March 19th and April 25th of 2020. The data were extracted from EPIC electronic medical record system using slicer dicer extraction tool. Our selection criteria include patients who tested positive for COVID-19 infection with pneumonia on imaging studies, who also had inflammatory markers documented during their admission period reducing the number to 235.

Categorical variables were presented as n (%) while continuous variables were reported as means ± SD or median (interquartile range, IQR) as appropriate. The differences between patients who died and those who survived were compared using chi-square, Fisher’s exact, Student t-test or Wilcoxon rank-sum test as appropriate. Patients were also compared in severe and non-severe categories using ICU admission as the severity factor. D-dimer levels were divided as mild < 2,000 ng/mL, moderate 2,000-3,000 ng/mL and severe >3,000 ng/mL.

All statistical analyses were performed using SAS 9.4 (SAS Institute Inc. Cary, NC) and a P-value < 0.05 was considered statistically significant.

## Results

Of the 235 COVID-19 positive patients included in this study, 35% died on admission by the end of this study, data shown in Table 1. Patients that died had a higher mean age compared to survivors (69.5 ± 13.6 years vs 63.8 ± 15.2 years, P = 0.004). The study population was predominantly non-Hispanic black with no statistically significant difference between the groups by race and/or ethnicity in terms of mortality (P = 0.69) and hospital length of stay (P = 0.23). 

**Table 1:** Demographic, and clinical characteristics. Values are reported as N (%), mean ± SD or median (interquartile range). CRP: C-reactive protein; INR: international normalized ratio; PTT: prothrombin time; LDH: lactate dehydrogenase; BMI: body mass index, kg/m^2^; VTE: venous thromboembolism; DVT: deep vein thrombosis; COPD: chronic obstructive pulmonary disease; NIPPV: non-invasive positive pressure ventilation lung disease - included COPD, asthma and interstitial lung disease malignancy - included all malignancies, both active and those with prior history of same.

	Survivors (N = 152)	Non-survivors (N = 83)	P value
Mean age, years	63.8±15.2	69.5±13.6	0.004
Obesity (BMI ≥30)	53(37.3)	26(35.1)	0.75
Female	61(40.1)	27(32.5)	0.25
Ethnicity/race, N (%)			
Hispanic	48(31.6)	26(31.3)	0.69
Non-Hispanic Black	90(59.2)	45(54.2)	
Non-Hispanic White	3(2.0)	2(2.4)	
Other	4(2.6)	5(6.0)	
Unknown	7(4.6)	5(6.0)	
Length of stay, days	8.0(5.0-15.0)	8.0(5.0-12.0)	0.23
Medical history			
Hypertension, N (%)	108(71.1)	57(68.6)	0.70
Diabetes, N (%)	56(36.8)	32(38.6)	0.80
Heart disease, N (%)	61(40.1)	29(34.9)	0.43
Lung disease, N (%)	30(19.7)	15(18.1)	0.76
Malignancy, N (%)	11(7.2)	10(12.1)	0.22
Smoker, N (%)	44(29.0)	23(27.7)	0.84
VTE, N (%)	6(4.0)	3(3.6)	>0.99
Inpatient outcomes			
D-dimer, ng/ml			0.002
Mild	95(62.5)	32(38.6)	
Moderate	11(7.2)	7(8.4)	
Severe	46(30.3)	44(53.0)	
INR, seconds	1.2(1.1-1.3)	1.3(1.1-1.5)	0.29
PTT, seconds	27.1(13.9-33.3)	22.1(15.0-30.0)	0.73
Ferritin, ng/ml	835.0 (441.0-1,550.0)	1153.0 (602.0-1,901.0)	0.80
CRP, mg/dl	10.6(5.1-20.9)	20.3(11.4-32.7)	<0.0001
LDH, U/L	454.0(322.0-628.5)	681.0(500.0-838.0)	<0.0001
Procalcitonin, ng/ml	0.3(0.1-1.1)	1.5(0.5-7.7)	<0.0001
DVT prophylaxis	124(81.6)	73(88.0)	0.20
Developed VTE	6(4.0)	1(1.2)	0.43
Intubation	28(18.4)	43(51.8)	<0.0001
NIPPV	2(1.3)	2(2.4)	0.62
ICU Admission	36(23.7)	46(55.4)	<0.0001

Hypertension was the main comorbidity, but no significant difference was observed between those who died and those who survived (68.6% vs 71.1%, P = 0.70). Diabetes mellitus, heart disease (heart disease was grouped as patients having arrhythmia, congestive heart failure (CHF) and coronary artery disease (CAD)) and cigarette smoking were also prominent comorbidities in this population but no significant difference in mortality was observed between the two groups (P = 0.80 vs 0.43 vs 0.84, respectively).

Laboratory findings significantly differed between the patient groups by D-dimer levels, C-reactive protein (CRP), lactate dehydrogenase (LDH), and procalcitonin in terms of the likelihood of intubation and ICU admission. There is a significant difference in the D-dimer levels in patients who survived when compared to those who died (P = 0.002). Compared to patients with mild D-dimer levels, patients with severe D-dimer levels are significantly more likely to die (OR = 2.84, 95% CI: 1.60-5.05), while those with moderate levels do not statistically differ (OR = 1.89, 95%CI: 0.68-5.29) (Table 1), Patients who died also showed significantly higher median CRP, LDH, and procalcitonin levels (Table 1). A higher proportion of patients who died were admitted to the ICU (23.7% vs 55.4%, P < 0.0001) and/or intubated (18.4% vs 51.8%, P < 0.0001) (Table 1). 

## Discussion

SARS-CoV-2 is a single-stranded RNA virus belonging to the family of Coronaviruses. The clinical characteristic of patients admitted to the hospital with COVID-19 infection has been described in multiple studies [6,7]. In our study, the key epidemiological findings include patient profile of non-Hispanic black males who were above the age of 60 years, with co-morbidities such as hypertension, heart disease, cigarette smoking and diabetes mellitus. These co-morbidities were significant risk factors for hospitalization but not mortality or length of stay.

Prior studies have expressed a disproportionate impact of COVID-19 infection on minority populations [8,9], thus making this study amongst the few that have sought to investigate this key cohort. As data emerges, one of the key areas of debate amongst researchers is the definition of clinical severity and prognostic indicators of poor outcomes in this disease. Length of stay has been utilized in previous studies as a marker of poor outcome or as a means of measuring therapeutic efficacy [10]. For our patient population, length of hospital stay, was not statistically significant among survivors. However, it is worth mentioning that in our hospital, there are usually multiple factors, predominantly those related to socio-economic status (such as lack of insurance and immigration status), which impact and lead to the prolongation of a patient’s time to discharge. Discharge protocols in patients with COVID-19 infection also continued to evolve as the pandemic progressed, further impacting their length of stay. As such, in our opinion, it limits its utility as a reliable tool for measuring outcome. Our study utilized admission to the Intensive Care Unit (ICU) as a key marker of disease severity. Admission criteria to the ICU in our institution include patients who were intubated or those who had impending clinical deterioration or respiratory failure warranting closer observation. Admission to the ICU (23.7% vs 55.4%, P < 0.0001) and endotracheal intubation (18.4% vs 51.8%, P < 0.0001) were key predictors of mortality.

The pathophysiological mechanism of COVID-19 infection has been postulated to be related to systemic vascular endothelial inflammation, thus predisposing patients to an increased risk of thromboembolic events [1,4]. This systemic inflammatory state elicited by COVID-19 has been well documented, with severely elevated D-dimer levels (especially above 2,000 mg/dL) highlighted as a marker of clinical severity [11], though there is no well-validated data. Amongst our patients, we divided our D-dimer levels into mild (<2,000 mg/dL), moderate (2,000-3,000 mg/dL), and severe (>3,000 mg/dL). We observed that levels above 3,000 mg/dL is most predictive of poor outcomes. All inflammatory markers, except for ferritin, were noted to be statistically significant (Table 1). Other studies also reported the significance of the inflammatory response as a poor prognostic factor [11,12]. Our facility did not assess fibrinogen levels frequently. In our cohort of patients, approximately 7.6% developed VTE, and there was no significant statistical difference amongst survivors and non-survivors (Table 1). 

Few studies have looked at prognosticators of poor outcomes in varying patient populations across the world [12,13]. A nationwide analysis of 1,590 patients hospitalized with COVID-19 infection in China, demonstrated that fatal outcomes were more prevalent in individuals 65 years and older (similar to our study) and associated co-morbidities of coronary heart disease and cerebrovascular disease [12,13]. In addition, it was noted that, although hypertension was a risk factor for hospitalization, it had no impact on mortality. This is similar to our observation in which patients with underlying hypertension showed no significant difference in mortality (68.6% vs 71.1%, P = 0.70). In another study in France, age was not found to be a prognostic factor for poor outcome but this study, much like ours, reflected the significant inflammatory markers such as CRP levels being a predictor of poor outcome [12]. Interestingly, studies from China and here in the United States have highlighted obesity as a risk factor for disease severity [7,9,13]. However, in our study, we did not find obesity to be a significant indicator of poor outcome.

Study limitations

This study has several limitations. The relative homogeneity of our study population limits its generalizability; however, we believe that it still provides a key insight into one of the highest risk populations and serves as a means of comparison with other populations in the United States, Italy, China and other countries that have been severely impacted. Fibrinogen levels were not consistently evaluated in our cohort, however, the evidence behind which inflammatory marker is better is scarce.

## Conclusions

Our study demonstrated that patients who died had a significantly higher D-dimer (>3,000 mg/dL) when compared with survivors. Higher mean age was associated with increased mortality and admission to ICU and/or intubation. Furthermore, this study highlights healthcare outcomes disparity as described in previous studies. This does not seem to correlate with the disease process itself but may rather highlight pre-existing healthcare disparities that place specific patient populations at higher risk.
